# Acetyl-11-Keto-β-Boswellic Acid Attenuates Prooxidant and Profibrotic Mechanisms Involving Transforming Growth Factor-β1, and Improves Vascular Remodeling in Spontaneously Hypertensive Rats

**DOI:** 10.1038/srep39809

**Published:** 2016-12-23

**Authors:** Peijin Shang, Wenxing Liu, Tianlong Liu, Yikai Zhang, Fei Mu, Zhihui Zhu, Lingfei Liang, Xiaohu Zhai, Yi Ding, Yuwen Li, Aidong Wen

**Affiliations:** 1 Department of Pharmacy, Xijing Hospital, Fourth Military Medical University, Xi’an, 710032, China

## Abstract

Vascular remodeling is an important complication of hypertension with oxidative stress-related profibrotic pathways involved. The transforming growth factor β1 (TGF-β1) has been shown to be a potential target of vasoprotection, and has multiple roles in vascular remodeling. Acetyl-11-Keto-β-Boswellic Acid (AKBA) is one of the active principles of Boswellic acids, and shows antioxidant activity in many diseases. The study is to determine effects of AKBA on systemic oxidative stress of hypertension and vascular remodeling. In the experiments, spontaneously hypertensive rats (SHR) were used. And *in vitro*, fibroblast was pretreated with AKBA before Ang II stimuli. In the results, treatment of AKBA markedly reduced oxidative stress, and decreased vascular remodeling by restoring vascular wall parameters and improving vascular reactivity. AKBA dramatically reduced TGF-β1 and Smad3 expression, as shown in immunofluorescence and immunohistochemistry. In cultured fibroblast, AKBA decreased intracellular ROS levels. Cell viability and proliferation, as well as migration were inhibited by AKBA. Additionally, treatment of AKBA significantly decreased TGF-β1 secretion in culture supernatant. Expression of TGF-β1, Smad3, P-Smad3 and Smad7 were also decreased by AKBA in fibroblast. In conclusion, AKBA is able to attenuate oxidative stress and profibrotic mechanisms, and improve vascular remodeling in hypertension through TGF-β1/Smad3 pathway.

Vascular remodeling, a non-ignorable part of vasculature pathological changes, is considered as a major risk of progressive cardiovascular diseases^1^. Initially it is functional, compensatory and adaptive^2^. However, chronic vascular remodeling aggravates vascular resistance and lumen narrowing, impairs vasodilatation and depletes compensative capacity of entire vasculature^3^, leading to severe cardiac, brain, kidney and other secondary damages^4^,^5^. Vascular remodeling even occurs before hypertension in young SHR aged 3 to 4 weeks^6^, indicating vascular remodeling may be one of key clues of incident hypertension^7^. Although present agents control blood pressure well, the nontoxic agents for vasoprotection are weakly effective but still necessary. It has been proved that excessive reactive oxygen species (ROS) are closely related to vascular pathologies^8^. Vascular wall is particularly vulnerable to oxidative damage. Thus, agents with antioxidant effect are used for prevention and treatment of vascular remodeling in hypertension.

Acetyl-11-Keto-β-Boswellic Acid (AKBA) is a pentacyclic triterpene compound from plant *Boswellia serrate* gum resins^9^, one of the most potent active principles. B. serrate extract has shown antioxidant effect for clinical use with good tolerability^10^, and recently it is found in our laboratory endothelial protection^11^, neuroprotection^12^, vasoprotection^13^, and regulation of vascular responses to inflammation^14^. It is also revealed that boswellic acids markedly decrease transforming growth factor-β1 (TGF-β1) and TGF-β1-induced pulmonary fibrosis^15^, and notably prevent colonic fibrosis through TGF-β1/Smad3 pathway^16^. Glycyrrhizin, the similar pentacyclic triterpene compound, has attenuated pulmonary vascular remodeling, as is reported^17^. Asiatic acid can alleviate cardiovascular remodeling due to its antioxidant effect^18^, and inhibit cardiac hypertrophy by blocking TGF-β1 pathway^19^. AKBA has similar structure and activity with Asiatic acid^20^. Based on the above studies, it is hypothesized that AKBA may also be beneficial for vascular remodeling in hypertension by blocking fibrotic TGF-β1 pathway.

TGF-β1 is one of key growth factors in vascular remodeling and formation of hypertension^21^,^22^. It phosphorylates subordinate receptors and transducers, especially canonical Smads pathway, and induces hundreds of genes expression^23^. While, Smad3 is reported the most relevant to vascular remodeling in this process^24^, making it a prime target for protection against vascular dysfunction. Over-activation of TGF-β1/Smad3 induces extracellular matrix (ECM) accumulation, fibrillar collagens deposition and elevated vascular cells viability, proliferation and migration, and ultimately results in vascular structural and functional alterations^25^. On the other hand, activation of dimer TGF-β1 is partially modulated by ROS^26^. The therapeutic effect of attenuating oxidative stress and preventing TGF-β1 during vascular remodeling in hypertension has been empirically proved^27^,^28^,^29^.

Therefore, in this study, it is hypothesized that AKBA may protect the vascular from remodeling in essential hypertension. The underlying mechanism of vasoprotection probably is associated with its good antioxidant effect, and thus inhibits over-activation of TGF-β1/Smad3 pathway. Vascular remodeling and profibrotic processes *in vivo* and *in vitro* are assessed.

## Results

### Effects of AKBA on systolic blood pressure, hemorheology and vascular contractility

SHR manifested higher levels of systolic blood pressure (SBP) at age of 7 weeks and continuously elevated in weeks ahead, and AKBA had no modification of blood pressure (see Supplementary Fig. S1). Same as hemorheology, AKBA had no effect on the whole blood viscosity (see Supplementary Table S1). Meanwhile, biochemical detection showed that SHR was challenged with higher vascular contractility that manifested with increased Ang II and decreased NO levels. However, EPI level remains normal. AKBA (20 mg/kg and 40 mg/kg) effectively attenuated vascular contractility through restoring Ang II and NO levels compared with SHR group (Table 1). The results indicated that AKBA exerted vasoprotection and decreased vascular contractility.

### AKBA attenuated oxidative stress *in vivo*

SOD, GPx bioactivities and MDA levels were measured in blood samples and vascular tissues (Table 2). SOD and GPx bioactivities of vascular tissue were decreased in SHR group compared with WKY group. AKBA treatment effectively restored SOD and GPx bioactivity. MDA level, an end product of lipid peroxidation, was increased in vascular homogenates of SHR compared with WKY. An evident reduction of MDA level was observed in AKBA treated-groups (20, 40 mg/kg) compared with SHR group. Consistently, AKBA treatment notably increased SOD, GPx bioactivity and decreased MDA levels in blood samples compared with SHR group.

### AKBA attenuated vascular remodeling of SHR

**Figure 1:**
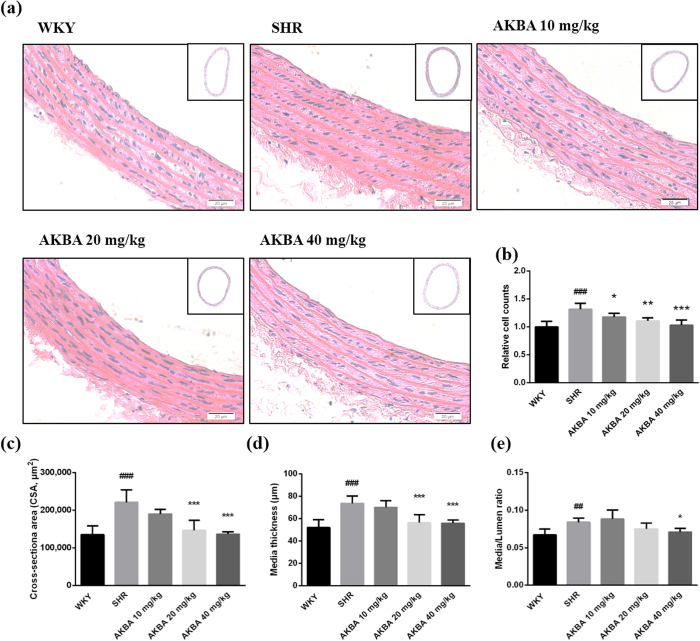
AKBA restored vascular morphological alterations. (**a**) Representative photographs of aortic samples (×200) stained by hematoxylin and eosin. Vascular parameters for assessment of vascular remodeling: (**b**) relative vascular cell number, (**c**) cross-sectional area, (**d**) media thickness and (**e**) Media/Lumen ratio. The data are represented as mean ± SD (n = 5). ^##^P < 0.01, ^###^P < 0.001 vs. WKY; *P < 0.05, **P < 0.01, ***P < 0.001 vs. SHR.

H&E staining showed that AKBA greatly improved thoracic aorta morphological alterations. Intact vascular rings under relaxation were photographed (see Supplementary Fig. S2) and the vascular parameters were measured with computerized image processing system. Local amplification of vascular walls was shown in Fig. 1a. Increased vascular cell number was found in SHR group. And AKBA significantly decreased cell number in a dose-dependent manner (Fig. 1b). In addition, vascular CSA, media thickness (M) and M/L ratio were significantly increased in SHR compared with WKY group. Treatment of 20, 40 mg/kg AKBA significantly decreased CSA (Fig. 1c) and media thickness (Fig. 1d), whereas, M/L ratio was significantly decreased only in 40 mg/kg AKBA treated-group (Fig. 1e).

### AKBA reduced collagen deposition *in vivo*

**Figure 2:**
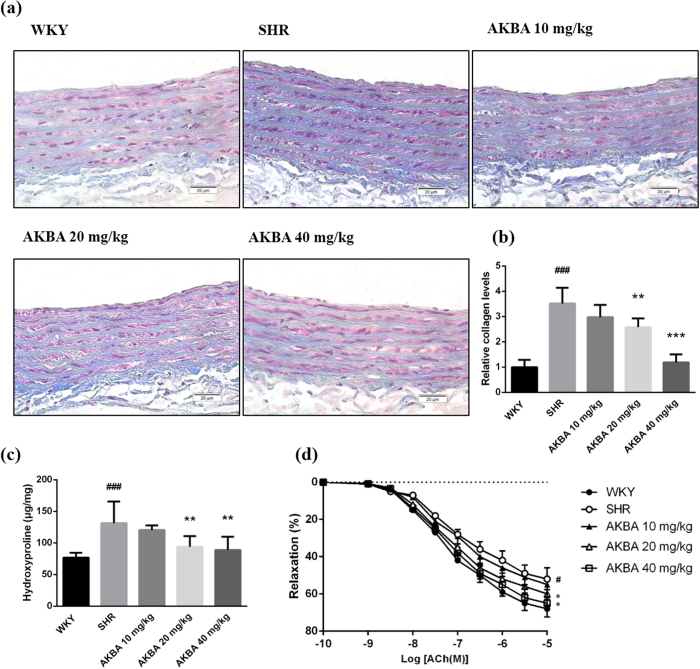
AKBA attenuated collagen deposition and restored vascular reactivity. (**a**) Representative photographs of Masson stained aortas (×200). (**b**) Semi-quantitative analysis of the mean density of the blue areas of Masson staining. (**c**) Hydroxyproline levels in vascular tissues. (**d**) Vascular relaxation. The results show that AKBA significantly decreases collagen deposition in vascular walls of hypertensive rats, and restores vascular reactivity. The data are represented as mean ± SD (n = 5). ^#^P < 0.05, ^###^P < 0.001 vs. WKY; *P < 0.05, **P < 0.01, ***P < 0.001 vs. SHR.

Collagen deposition is another influential characteristic of vascular remodeling. AKBA obviously reduced collagen deposition, as shown in the blue area in Masson staining of vascular walls (Fig. 2a). According to semi-quantitative analysis, compared with WKY group, collagen deposition in SHR group was dramatically enhanced. Treatment of AKBA decreased collagen deposition in vascular wall in a dose-dependent manner (Fig. 2b). Likewise, AKBA effectively decreased hydroxyproline levels compared with SHR group (Fig. 2c). The results further confirmed that AKBA effectively decreased vascular collagen expression and deposition in vascular walls.

### AKBA restored vascular reactivity of SHR

The concentration-relaxation response curve to acetylcholine (Ach) showed that vascular relaxation in SHR was weakened. Compared with WKY group, thoracic aortic rings that were pre-contracted by 10^−6^ mol/L phenylephrine from SHR showed declining vascular relaxant response to acetylcholine (Ach) (P < 0.01). While, the Ach-mediated vascular relaxation was significantly augmented in AKBA treated-groups (20, 40 mg/kg) compared with SHR group (Fig. 2d). The finding suggested that AKBA could prevent hypertension-induced impairment of vasodilatation.

### AKBA decreased TGF-β1/Smad3 expression *in vivo*

**Figure 3:**
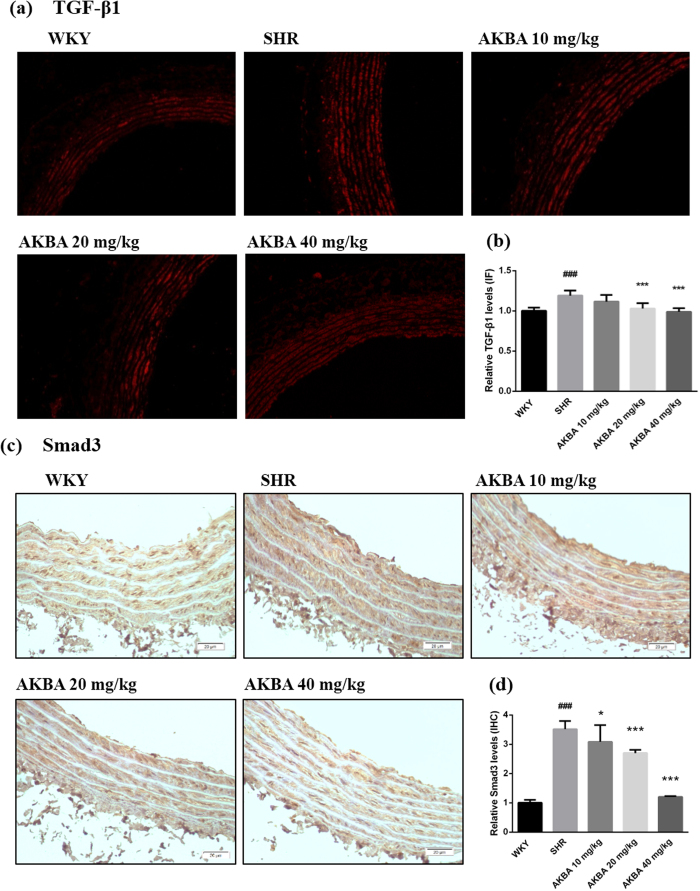
AKBA decreased TGF-β1/Smad3 in hypertensive rats. (**a**) Vascular TGF-β1 expression was assessed by immunofluorescence. (**c**) Smad3 expression was assessed by immunohistochemistry. Semi-quantitative analysis of (**b**) TGF-β1 and (**d**) Smad3 demonstrates AKBA’s potential to decrease TGF-β1 and Smad3 proteins expression in vascular walls. The data are represented as mean ± SD (n = 5). ^###^P < 0.001 vs. WKY; *P < 0.05, ***P < 0.001 vs. SHR.

To clarify whether TGF-β1/Smad3 pathway was involved in the vascular protective effects of AKBA, their expressions in vascular walls of hypertensive rats were detected by immunofluorescence and immunohistochemistry respectively. The results showed that expression of TGF-β1 and Smad3 in SHR were increased compared with WKY group, and AKBA treatment notably hindered expression of TGF-β1 and Smad3 (Fig. 3).

### AKBA decreased intracellular ROS *in vitro*

**Figure 4:**
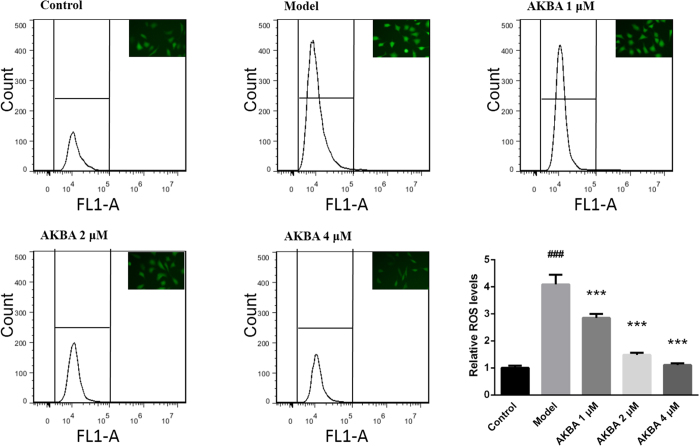
AKBA decreased intracellular ROS production in fibroblast. Intracellular ROS *in vitro* was assessed by flow cytometer. Quantitative analysis shows that AKBA decreases ROS due to inducing effect of Ang II in a dose-dependent manner. The data are represented as mean ± SD (n = 5). ^###^P < 0.001 vs. control group; ***P < 0.001 vs. model group.

Fibroblasts stimulated by Ang II significantly increased intracellular ROS production compared with the control group. AKBA intervention significantly decreased ROS secretion in a dose-dependent manner (Fig. 4).

### AKBA decreased fibroblast viability and proliferation and inhibited excess migration

**Figure 5:**
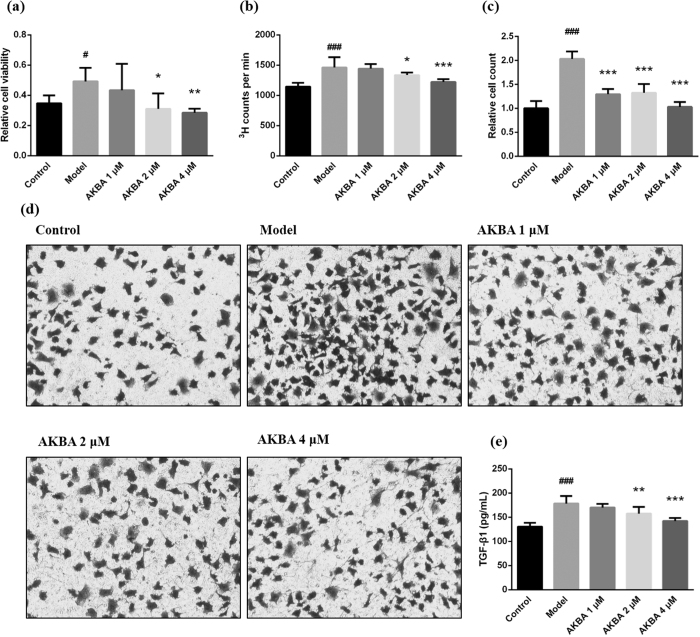
AKBA decreased fibroblast viability, proliferation and migration, and lessened TGF-β1 secretion. (**a**) The results of cell viability assessment by MTT assay prove that AKBA can notably decrease fibroblast viability under inducing effect of Ang II. (**b**) ^3^H-thymidine incorporation assay shows that AKBA effectively decreases fibroblast proliferation induced by Ang II. (**c**) Quantitative analysis shows that AKBA is able to prevent excess vascular fibroblast migration triggered by Ang II stimuli. (**d**) Cell migration was assessed by Transwell assay. (**e**) TGF-β1 secretion in culture supernatant was measured by ELISA. The results shows that AKBA significantly decreases TGF-β1 content induced by Ang II. The data are represented as mean ± SD (n = 5). ^#^P < 0.05, ^###^P < 0.001 vs. control group; *P < 0.05, **P < 0.01, ***P < 0.001 vs. model group.

Vascular adventitial fibroblast viability, proliferation and migration are another important pathological processes to vascular remodeling. Compared with control group, Ang II stimulated fibroblast viability (Fig. 5a) and proliferation (Fig. 5b). Treatment of AKBA (20, 40 mg/kg) significantly decreased fibroblast viability and proliferation. Moreover, AKBA effectively decreased excess fibroblast migration that was increased in model group (Fig. 5c,d).

### AKBA decreased TGF-β1/Smad3 pathway *in vitro*

**Figure 6:**
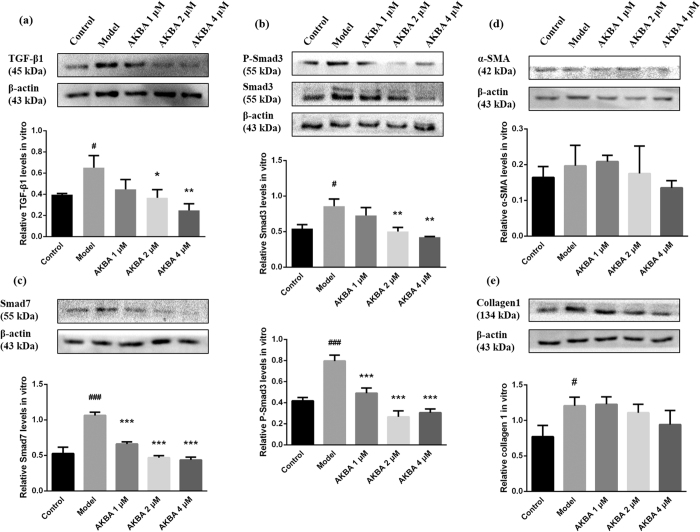
AKBA inhibited TGF-β1/Smad3 pathway *in vitro*. TGF-β1/Smad3 and several proteins in cell extracts involved in this pathway were assessed by western blot *in vitro*. AKBA effectively decreased (**a**) TGF-β1, (**b**) Smad3 and P-Smad3, (**c**) Smad7 levels. The conclusion is that AKBA can notably inhibit TGF-β1/Smad3 pathway, but have no modification effect on (**d**) α-SMA and (**e**) collagen-1 expression within 24 h treatment *in vitro*. The data are represented as mean ± SD (n = 5). ^#^P < 0.05, ^###^P < 0.001 vs. control group; *P < 0.05, **P < 0.01, ***P < 0.001 vs. model group.

To further identify whether TGF-β1 is involved in the protective effects of AKBA, TGF-β1 secretion was analyzed by ELISA and western blot *in vitro*. The data showed that TGF-β1 secretion was increased in culture supernatant of model group compared with control group (P < 0.001), whereas, AKBA treatment effectively decreased TGF-β1 secretion in culture supernatant (Fig. 5e). Consistently, western blot showed Ang II mediated expression of TGF-β1 was reduced by AKBA treatment (Fig. 6a). In model group, expression of Smad3/P-Smad3, as a pair of cytoplasm signal transducers of TGF-β1 were increased. And AKBA effectively decreased Smad3 and P-Smad3 expression (Fig. 6b). Smad7 is one of Smad inhibitors. The results showed that expression of Smad7 in model group was increased, and AKBA significantly decreased Ang II triggered Smad7 expression (Fig. 6c). No significant differences of α-SMA (Fig. 6d) and collagen-1 (Fig. 6e) were found between model group and AKBA treated groups.

## Discussion

As an important complication of hypertension, vascular remodeling has been increasingly regarded as a major risk of cardiovascular diseases. Oxidative damage plays a critical role in this process^8^,^30^. Gene transfer of catalase can reduce ROS production and ameliorate vascular remodeling, as is found^31^. Based on good antioxidant effect, multiple compounds or drugs have therapeutic effect on vascular remodeling in different models^32^. Analogously, the protective effect on vascular wall has been highlighted by down-regulating expression of profirotic factor TGF-β1^33^, which could be activated by overexpressed free oxygen radicals. It is found in similar studies that sustained H_2_O_2_ stimulates TGF-β1 synthesis and transcription of subordinate collagens and other ECM proteins, which could be restored by pretreatment of catalase and anti-TGF-β1 antibody^34^. In another word, drugs inhibiting TGF-β1 pathway or in combination with antioxidant effect may have potential vasoprotection in vascular remodeling. Pentacyclic triterpene compounds structurally similar to AKBA, such as glycyrrhizin and Asiatic acid, have been reported therapeutic effects on cardiovascular remodeling and fibrosis^17^,^18^,^19^. Accordingly, AKBA may also have potential implication in treating vascular remodeling of hypertension, without proper mechanisms, however. This study first focuses on this new application of AKBA in vascular remodeling of hypertension. The results suggest that AKBA effectively inhibits profibrotic factors TGF-β1/Smad3 expression and activation, and prevents vascular remodeling in association with its antioxidant effect.

Primarily, it is found that hypertensive rats exhibit aortic hypertrophy, increased collagen deposition and decreased vascular reactivity, in association with increased profibrotic factor TGF-β1 expression and activity. These processes may account for oxidative stress. AKBA is characterized by superoxide scavenging properties through Nrf2/HO-1 pathway^12^. In line with these studies, it is discovered that treatment of AKBA decreases oxidative stress in blood and vascular walls. AKBA markedly increases SOD and GPx bioactivity and decreases MDA levels in both of serums and vascular homogenates (Table 2). And none significant side effects on renal function, body weight and growth index were observed (see Supplementary Table S2 and Fig. S3). Followed, intracellular ROS was induced by Ang II in fibroblast (Fig. 4). Treatment with AKBA markedly decreases ROS level in a dose-dependent manner, which further confirms antioxidative activity of AKBA in hypertension. However, AKBA did not directly affect higher blood pressure (see Supplementary Fig. S1) and abnormal hemorheology (see Supplementary Table S1).

Additionally, vascular contractility is measured on the basis of vasodilator and vasoconstrictors levels in blood. AKBA markedly restores Ang II and NO secretion in blood samples (Table 1), which suggests that vascular contractility is decreased. Morphologically, expanded vascular rings has been shown in Supplementary Fig. S2. Vascular cell number, CSA, M and M/L ratio in SHR are significantly increased, which directly indicates that hypertensive rats are suffered with severe vascular remodeling. AKBA notably attenuates vascular remodeling and restores these parameters to comparative normal levels (Fig. 1).

Collagen deposition is another prominent feature of vascular remodeling or vascular fibrosis. It is modulated by TGF-β1/Smad3 pathway and shows negative effects on vascular tone and structure. As shown in Fig. 2a and b, AKBA dramatically decreases collagen deposition in vascular walls of SHR. And also, AKBA is able to explicitly decrease hydroxyproline levels in vascular walls (Fig. 2c). Furthermore, the experiment on vascular reactivity demonstrates that AKBA can effectively increase vascular relaxation induced by increasing concentration of Ach in a dose-dependent manner (Fig. 2d). Drugs with antioxidant effect have the capacity to inhibit vascular remodeling or tissue fibrosis^28^. In the study, antioxidant AKBA blunts hypertension-induced vascular remodeling. *In vitro* studies, increased vascular fibroblast viability, proliferation and migration are considered as another important processes and features of vascular remodeling^35^, which are modulated by TGF-β1 pathway. Activated fibroblast may secret multiple cytokines, enzymes and chemokines that influence cell proliferation, differentiation and migration, so as to form a feedback loop and trigger vascular remodeling at early stage of hypertension^36^,^37^. AKBA effectively decreases fibroblast viability, proliferation and migration (Fig. 5a–d).

As referred above, hypertension induces multiple structural alterations of the arterial wall with excessive ECM accumulation and collagen deposition, elevated vascular cell viability, proliferation and migration. This vascular remodeling process is linked to the activation of several intracellular signaling pathway of growth factors such as TGF-β1^38^,^39^. Excessive TGF-β1 plays a causal role in progressive aortic enlargement and contributes to aortic aneurysm^40^. According to the observation, soluble guanylate cyclase notably decreases TGF-β, so as to inhibit experimental fibrosis and vascular diseases^41^. TGF-β1 combines with receptors on membrane and phosphorylates cytoplasmic signal transducers R-Smads (Smad2/3), which shuttle into the nuclear and combine with DNA-binding co-factors^42^,^43^, and then selectively bind to specific sequence of target genes and modulate potential hundreds of genes expression^44^. Thus, TGF-β1 modulates cell proliferation, apoptosis and migration, and elicits various biological responses selectively and accurately. While canonical Smad3 pathway mainly modulates ECM accumulation^24^, as is found. However, this process can be stopped by Smad7 in cytoplasm, which promotes R-Smads complex ubiquitination or depolymerization and thus terminates signaling conduction^42^. Consistently^45^,^46^, AKBA notably decreases TGF-β1 and Smad3 expression in vascular walls of SHR (Fig. 3), as well as in cell culture of rat vascular fibroblast with Ang II stimuli (Figs 5d and 6a,b). And P-Smad3 and Smad7 *in vitro* are also retreated by AKBA treatment (Fig. 6b and c), but no significances were found in the expression of collagen 1 and α-SMA, a biomarker of myofibroblast modulated by TGF-β1/Smad3, between model and AKBA treated groups in fibroblast upon Ang II stimuli (Fig. 6d and e). These results indicate a comparatively active condition of TGF-β1/Smad3 pathway in early stage of hypertensive models *in vitro*.

In this study, AKBA inhibits profibrotic mechanisms, and exerts therapeutic effects *in vivo* and *in vitro*. The findings provide novel mechanistic insights about the beneficial effects of AKBA in hypertension without reducing blood pressure. The new application of AKBA on vascular remodeling make such a natural plant-extracted compound a promising therapeutic agent on vascular remodeling particularly. Additionally, AKBA has the potential to be an alternative or supplementary therapeutic strategy for the conventional treatment of vascular remodeling in multiple cardiovascular diseases.

However, several issues still need to be addressed in future studies. For example, whether other factors or signal pathway simultaneously contribute to the protective effects of AKBA, typically including microRNA^47^,^48^, NF-κB and MMPs^13^,^14^. And blood pressure and intracellular ROS production should be observed more precisely. In addition, in the three-layered structure of vascular wall, each layer and each cell type has specific biochemical and functional characteristics, thus, the possible protective effects on other cell lines related to vascular remodeling remains to be clarified.

Collectively, in this study, it is firstly demonstrated that AKBA could effectively decrease vascular remodeling in spontaneously hypertensive models, and inhibit Ang II induced fibrotic pathways involvement of TGF-β1/Smad3. And the protective effects are largely related to the good antioxidant effect of AKBA. To sum up, this study provides evidence about potential implication of AKBA on blocking prooxidant and profibrotic factors, mainly referring to TGF-β1/Smad3, to prevent or reverse vascular remodeling in hypertension.

## Materials and Methods

### Animals and sample preparation

The experiments are approved by Ethics Committee of Animal Experimentation of the Fourth Military Medical University (Xi’an China), and accord with Institutional Guidelines for Care and Use of Laboratory Animals. 20 spontaneously hypertensive rats (SHR, male, 6w) and 5 Wistar-Kyoto (WKY) rats (male, 6w), body weight 140–160 g, were obtained from the Laboratory Animal Center of the Fourth Military Medical University. The rats were kept at room temperature 23 ± 2 °C with free access to water and chow (12 h light/dark cycle). SHR were divided into 4 groups randomly (n = 5): vehicle-treated SHR group (SHR), 10 mg/kg AKBA-treated SHR group (AKBA 10 mg/kg), 20 mg/kg AKBA-treated SHR group (AKBA 20 mg/kg), 40 mg/kg AKBA-treated SHR group (AKBA 40 mg/kg), and vehicle-treated WKY group (WKY) (n = 5) was defined as control group. AKBA (reagent grade, purity, Santa Ana, CA) was diluted by deionized water administrated by gavage for 8 weeks. After that, all of the rats were anesthetized by intraperitoneal injection of chloral hydrate (10%, 3 ml/kg) and sacrificed by exsanguination. Blood samples were collected. Thoracic aortas perfused with 10 ml normal saline, and fixed by 10 ml 4% paraformaldehyde (pH 7.4) under physiological pressure. Samples were dissected carefully in normal saline upon ice-bath. Next, tissues were fixed in 4% phosphate-buffered paraformaldehyde, pH 7.4, and embedded in paraffin blocks.

### Systolic blood pressure, hemorheology and vascular contractility

After one week adaptive training, systolic blood pressure (SBP) was measured via tail-cuff method weekly during waking hours (BP-2010A, Softron Beijing Biotechnology Co., Ltd, Beijing, China). SBP was measured three times under rest state, and then average values were obtained. Blood samples were collected 8 weeks later. Within 4 hours, whole blood viscosity was detected. Vascular contractility was preliminarily measured by relevant vasoactive substances in serum. ABC-ELISA kits (Westang Biotechnology Co., Ltd, Shanghai, China) were used to detect angiotensin II (Ang II) and epinephrine (EPI) levels in serum. In addition, vascular relaxant factor, nitric oxide (NO) in serum, was measured by NO kit (Beyotime Biotechnology, China) with Griess reagent. All of the experimental procedures were followed in accordance with the rules designated by the manufacturer.

### Assessment of vascular oxidative stress

After the rats were sacrificed, blood samples and thoracic aortas were collected. Vascular tissues were weighted equally. Blood samples and vascular homogenates were centrifuged at 3000 rpm for 10 min, and the supernatant obtained was used in the following experiments. Superoxide dismutase (SOD), glutathione peroxidase (GPx) activity and malondialdehyde (MDA) content in serum and vascular tissues were detected by respective kits (Jiancheng Bioengineering institute, Nanjing, China). All of experimental procedures were followed in accordance with the rules designated by the manufacturer.

### Morphometric analysis and composition of the vascular wall

Thoracic aortas were deparaffined and 4 μm-thick slices were stained with haematoxylin and eosin (H&E) and Masson stain. The intact vascular rings were photographed by microscope (Olympus, Japan) under relaxation. Thoracic aorta cross-sectional area (CSA) was estimated by subtracting lumen area (A_i_) from external area (A_e_). The external diameter (D_e_) and internal diameter (D_i_) were calculated via 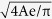 and 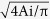 respectively, and media thickness (M) was calculated by (D_e_ − D_i_)/2. And then M to lumen ratio (M/L) was calculated. The relative vascular smooth muscle cell number in vascular media was measured by the computerized microscope system, independent of cell orientation, form and size of the nucleus. Masson staining was used to preliminarily evaluate collagen deposition in vascular walls. Stained vascular rings were photographed at 200× by microscope. Collagen deposition was assessed by the mean optical density of blue area. In addition, collagen expression was assessed by hydroxyproline assay. Vascular tissues (50 mg) were digested by hydrolysate at 95 °C for 20 min, and then centrifuged at 3500 rpm for 10 min. The supernatant was collected and operated, after hydroxyproline test kit protocol (Jiancheng Bioengineering institute, Nanjing, China).

### Study on vascular reactivity

Intact thoracic aortic rings (3 mm) were dissected and mounted in the organ chambers. Vascular rings were incubated for 30 min in Krebs buffer (consisting of (mM): NaCl 118, KCl 4.75, NaHCO_3_ 25, MgSO_4_ 1.2, CaCl_2_ 2, KH_2_PO_4_ 1.2, glucose 11) aerated with 95% O_2_ and 5% CO_2_ at 37 °C. The rings were stretched to 2 g of resting tension and equilibrated for 60 min. Vascular relaxation was assessed in response to increasing concentration of acetylcholine (Ach, 10^−10^ M to 10^−5^ M) after pre-contracted by 10^−6^ mol/L phenylephrine.

### Assessment of TGF-β1/Smad3 expression *in vivo*

Aortic sections were incubated with TGF-β1 antibody (Rabbit monoclonal to TGF-β1, 1:200, Bioss, bs-0086R) in dark humidified chambers for 1 h. And then anti-rabbit rhodamine conjugated secondary antibody was added and then washed by PBS 1 hour later. The pictures were taken by fluorescent microscope. The red fluorescence indicated TGF-β1 expression, and the intensity was analyzed by Image pro-plus 6.0. Aortic rings were incubated with 3% H_2_O_2_ at room temperature for 8 min to block endogenous peroxidase activity, and then rinsed with diluted water and soaked in PBS for 5 min. Then tissue sections were incubated with specific rabbit anti-Smad3 antibody (1:100, Abcam, ab40854) for 1 h at 37 °C in dark humidified chambers. The sections were washed 3 times with PBS, and then the added anti-rabbit HRP-conjugated secondary antibody took effect for 30 min at 37 °C. Smad3 expression was evaluated as the dark brown staining in the whole section, and the mean optical density was calculated by Image-Pro Plus 6.0.

### Cell cultivation

Rat vascular adventitia fibroblasts were purchased from Innovate Biotechnology (Ltd, Wuxi, China). Cells were cultured in Dulbecco’s Modified Eagle Medium (DMEM) /high glucose containing 10% fetal bovine serum (FBS) under humidified atmosphere with 5% CO_2_ at 37 °C, and culturing medium was replaced every two days. Cells (passages 3–6) were seeded onto cell plates. And the cells were divided into 5 groups as below: vehicle-treated group (control), 0.1 μM Ang II-treated group (model), 0.1 μM Ang II + 1 μM AKBA-treated group (AKBA 1 μM), 0.1 μM Ang II + 2 μM AKBA-treated group (AKBA 2 μM), and 0.1 μM Ang II + 4 μM AKBA-treated group (AKBA 4 μM). The cells reached 80–90% confluence 24 h later, and then they were starved for 24 h under serum-free medium. After that, each group was pretreated with different concentrations of drugs 1 h before Ang II (0.1 μM) stimuli, and cultured for another 24 h.

### Assessment of ROS production by flow cytometer

*In vitro*, oxidative stress was measured though measurement of intracellular ROS generation. The cells were planted in 6-well plates at a density of 10,000 per well with 1 mL culture medium. After treatment, DCFH-DA (10 μM) dissolved in serum-free medium was added and incubated for 30 min. The cells were collected and diluted with 200 μl PBS, and then detected by flow cytometer.

### Cell viability and proliferation assay

Fibroblast viability and proliferation were investigated by MTT and ^3^H-thymidine incorporation assays. Fibroblasts were planted in 96-well plates at a density of 3000 per well. 3-(4, 5-dimethyl-2-thiazolyl)-2, 5-diphenyl-2-H-tetrazolium bromide (MTT, 5 mg/mL) was dissolved in PBS. After 24 h treatment, cells were cultured with serum-free medium containing 20 μL MTT (0.5%) solution for 4 h, and the plates were washed by PBS 3 times. Thereafter, 200 μL DMSO was used to dissolve the purple precipitate, and the absorbency was detected at 570 nm. Fibroblast viability was defined as the relative absorbance. Similarly, cells were plated at 3000 per well in 96-well plates. Before 6 h of the end of the treatment, ^3^H-thymidine (2 μCi/mL) was added and cultured for 16 h. ^3^H-thymidine incorporation was detected by Geiger counter (Beckman LS-9800). Fibroblast proliferation was defined as ^3^H counts per min of each plate.

### Cell migration assay

Fibroblast migration was accessed by Transwell assay. The cells were disposed in upper chambers at a density of 3000 in 100 μL serum-free medium, and the lower ones were filled with 600 μL medium with corresponding drugs. The cells were fixed by ethyl alcohol for 5 min 14 h later, and then stained with 0.1% crystal violet for 10 min. After that, the cells remained in the upper ones were removed, and the migrated parts stained with purple were pictured.

### Assessment of TGF-β1 secretion by ELISA

The cells were planted in 96-well plates at a density of 3000 per well with 200 μL culture medium. After 24 h incubation, they were starved for 24 h. And then each plate was pretreated with corresponding concentration of AKBA 1 h before Ang II stimuli, and cultured for 24 h. The culture supernatant was collected. TGF-β1 secretion *in vitro* was tested by ELISA kit (LanPaiBio, ShangHai, China). The experimental procedures were followed in accordance with the rules designated by the manufacturer.

### Western blot assessment of TGF-β1/Smad3 pathway

The relevant protein levels of TGF-β1, Smad3, P-Smad3, Smad7, α-SMA and collagen-1 *in vitro* were measured by western blot. Cells incubated and treated in 6-well plates were lysed by 200 μL RIPA lysis buffer containing protease inhibitor (PMSF, 1 mM) and phosphatase inhibitor (TIANDZ 80809-1, 1%) on ice-bath for 30 min. And the lysate was centrifuged at 10000 rpm for 10 min at 4 °C, the supernatant was collected and the proteins were quantified by BCA protein assay kit (Beyotime Biotechnology, China). Membranes were incubated with primary antibodies (anti-TGF-β1, 1:1000, Abcam, ab64715; anti-Smad3, 1:1000, Abcam, ab40854; anti-P-Smad3, 1:400, Bioss, BS-3425R; anti-Smad7, 1:400, Boster, BA1399; anti-α-SMA, 1:400, Boster, BM0002; anti-collagen1, 1:400, Boster, BA0325) overnight at 4 °C and mouse monoclonal anti-β-actin (1:500, Sinipept, 41302M) was used for loading control. After that HRP-goat-anti-mouse antibody (1:1000, World Biotech, WH-004) was incubated with membranes of TGF-β1, α-SMA and β-actin for 1 h at room temperature; HRP-goat-anti-rabbit antibody (1:1000, CW-biotech, cw-0103) with Smad3, P-Smad3, Smad7 and collagen-1 respectively. Immunoblot were visualized with Bio-Rad and recorded by Bio-Rad detection system, and gray density analysis was completed with the Image J program.

### Statistical analysis

All the data involved are presented as mean ± standard deviation (SD) and analyzed by spss17.0 (SPSS Inc., Chicago, IL, USA). Statistic variables of all groups were compared with one-way classification ANOVA, followed by LSD-t test. The statistically significant level is regarded as P < 0.05.

## Additional Information

**How to cite this article**: Shang, P. *et al*. Acetyl-11-Keto-β-Boswellic Acid Attenuates Prooxidant and Profibrotic Mechanisms Involving Transforming Growth Factor-β1, and Improves Vascular Remodeling in Spontaneously Hypertensive Rats. *Sci. Rep.* **6**, 39809; doi: 10.1038/srep39809 (2016).

**Publisher's note:** Springer Nature remains neutral with regard to jurisdictional claims in published maps and institutional affiliations.

## Supplementary Material

Supplementary Information

## Figures and Tables

**Table 1:** Vasodilator and vasoconstrictors.

Groups	NO (μM/L)	Ang II (ng/mL)	EPI (ng/mL)
WKY	27.72 ± 6.10	2.17 ± 0.48	4.48 ± 1.06
SHR	13.38 ± 3.65^###^	4.36 ± 1.46^##^	5.17 ± 1.30
AKBA 10 mg/kg	16.38 ± 2.49	3.51 ± 1.09	5.10 ± 0.92
AKBA 20 mg/kg	21.76 ± 1.99^**^	2.94 ± 1.22^*^	4.18 ± 0.86
AKBA 40 mg/kg	24.46 ± 2.38^***^	2.31 ± 0.62^**^	4.84 ± 0.84

The data are represented as mean ± SD (n = 5). ^##^P < 0.01, ^###^P < 0.001 vs. WKY group; ^*^P < 0.05, ^**^P < 0.01, ^***^P < 0.001 vs. SHR group.

**Table 2:** Oxidative stress assessment in blood and vascular walls.

Groups	Serum			Thoracic aorta		
	SOD (U)	GPx (U)	MDA (nM/L)	SOD (U)	GPx (U)	MDA (nM/L)
WKY	202.83 ± 19.57	820.61 ± 97.59	7.01 ± 1.65	96.01 ± 7.47	362.71 ± 28.51	4.41 ± 0.50
SHR	130.90 ± 16.63^###^	636.04 ± 77.07^##^	11.52 ± 2.98^##^	58.39 ± 5.67^###^	297.53 ± 12.07^###^	6.85 ± 0.75^###^
AKBA 10 mg/kg	143.94 ± 14.34	688.59 ± 54.50	9.05 ± 2.29	69.27 ± 6.72^*^	313.36 ± 10.22	6.49 ± 0.45
AKBA 20 mg/kg	177.39 ± 18.64^**^	759.39 ± 54.28^*^	8.22 ± 1.48^*^	78.13 ± 8.32^**^	329.75 ± ± 7.47^**^	6.05 ± 0.44^**^
AKBA 40 mg/kg	199.06 ± 18.04^***^	812.73 ± 72.77^**^	7.79 ± 1.53^*^	92.91 ± 9.43^***^	346.72 ± 11.82^***^	5.31 ± 0.70^***^

The data are represented as mean ± SD (n = 5). ^##^P < 0.01, ^###^P < 0.001 vs. WKY group; ^*^P < 0.05, ^**^P < 0.01, ^***^P < 0.001 vs. SHR group.
