# Supplementary material for: Acetyl-11-Keto-β-Boswellic Acid Attenuates Prooxidant and Profibrotic Mechanisms Involving Transforming Growth Factor-β1, and Improves Vascular Remodeling in Spontaneously Hypertensive Rats
Source: Sci Rep. 2016 Dec 23;6:39809. doi: 10.1038/srep39809 (PMC5180224; doi:10.1038/srep39809)
Supplement: Supplementary Information [file srep39809-s1.pdf]

Supplementary materials:

**Acetyl-11-keto-β-boswellic acid attenuates prooxidant and profibrotic mechanisms involving transforming growth factor-β1, and improves vascular remodeling in Spontaneously Hypertensive Rats**

Running title: AKBA decreases TGF-β1 and vascular remodeling

Authors: Peijin Shang[a]‡, Wenxing Liu[a]‡, Tianlong Liu[a]‡, Yikai Zhang[a], Fei Mu[a], Zhihui Zhu[a], Lingfei Liang[a], Xiaohu Zhai[a], Yi Ding [a*], Yuwen Li[a*], **Aidong Wen[a*]**

‡: These authors contribute equally to this study.

*: Corresponding Authors: Yi Ding (dingyi.007@163.com), YuWen Li (liyuwenzs@gmail.com) and **AiDong Wen**

**(adwen-2004@hotmail.com)**

a: Department of Pharmacy, Xijing Hospital, Fourth Military Medical University

**Fig S1.** Systolic blood pressure. Compared with WKY group, spontaneously hypertensive rats (SHR) show higher systolic blood pressure (SBP) at 7 weeks of age that continuously elevates in weeks ahead, but AKBA has no effect on SBP, mean ± SD (n=5).

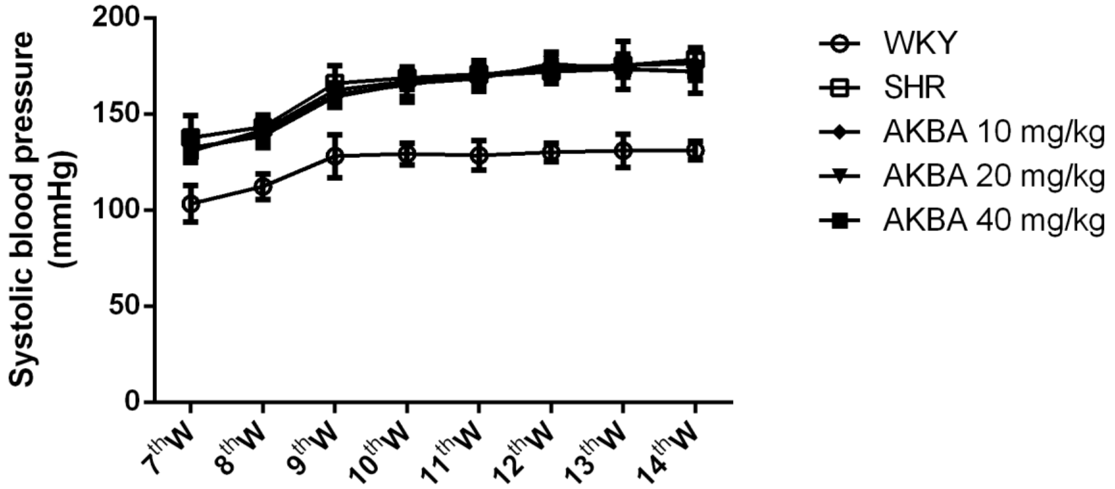

**Fig S2**. Intact vascular rings and vascular parameters. Intact vascular rings are photographed and vascular parameters are measured in computerized image processing system. Vascular external area, internal area, external diameter and internal diameter are calculated and analyzed. AKBA significantly decreases vascular hypertrophy and remodeling in SHR, and restores vascular morphology to comparative normal levels. Mean ± SD (n=5), $^{\#}P<0.05$ vs. WKY group, $^{*}P<0.05$ vs. SHR group.

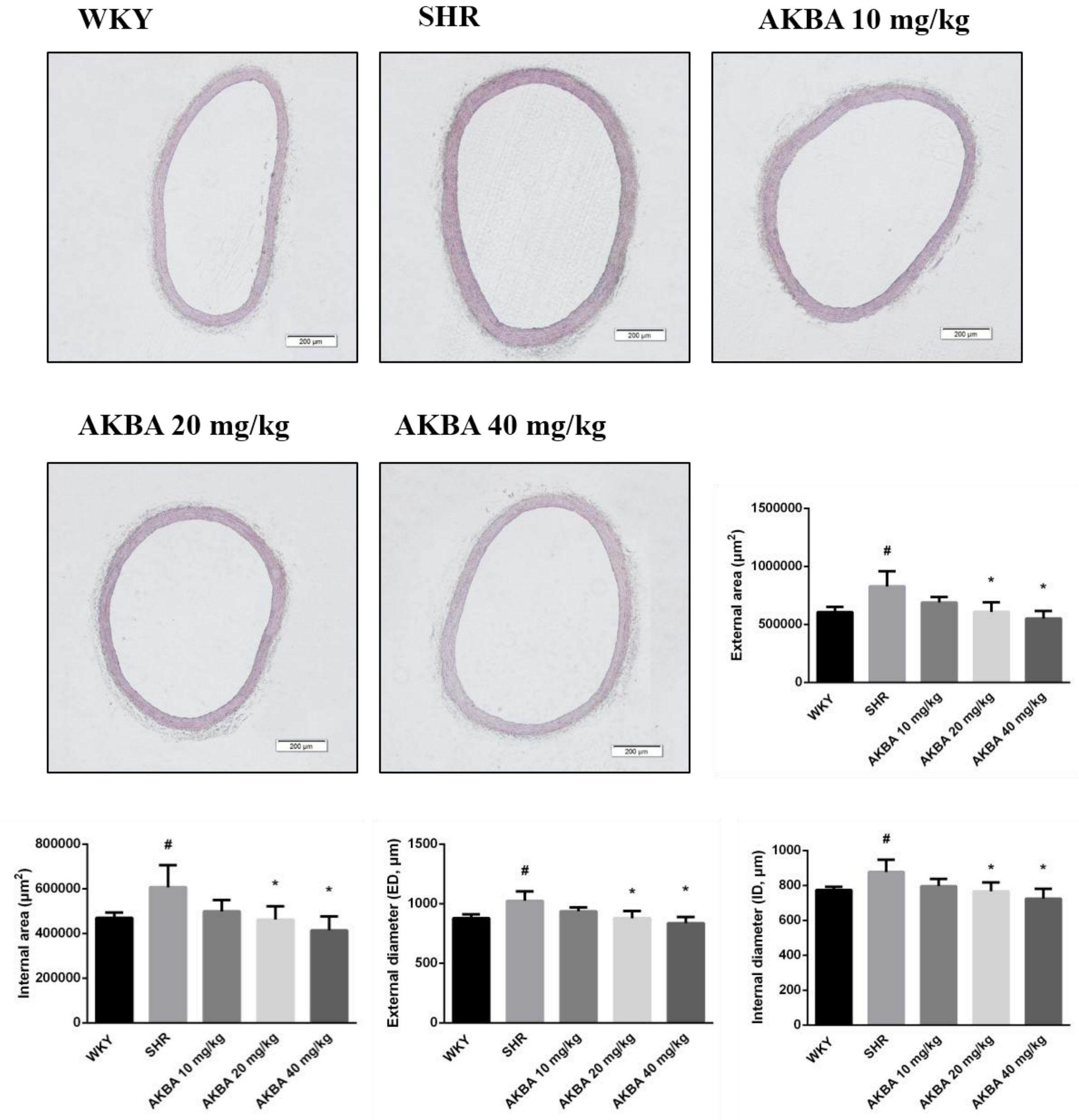

**Fig S3.** Body weight and growth index. The results shows that AKBA has no effects on body weight and growth index, calculated as (W14 –W6)/W6 (W14 =weight of 14w, W6=weight of 6w), mean ± SD (n=5).

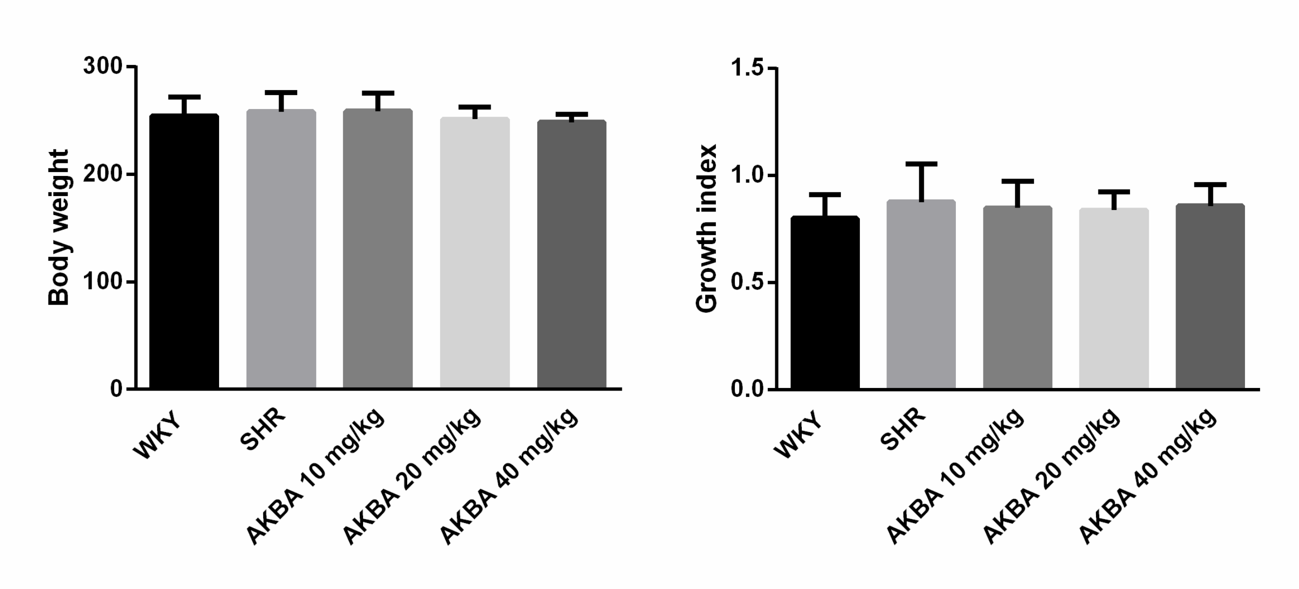

**Table S1** Hemorheology, whole blood viscosity

| Animal Groups | Shear rates (1/s) | | | |
| --- | --- | --- | --- | --- |
| | 1 | 5 | 30 | 200 |
| WKY | 19.83±2.98 | 8.72±1.18 | 4.99±0.60 | 3.73±0.42 |
| SHR | 26.21±1.26# | 10.90±0.34# | 5.91±0.38# | 4.26±0.40# |
| AKBA 10mg/kg | 25.21±1.42 | 10.92±0.45 | 5.88±0.18 | 4.24±0.12 |
| AKBA 20mg/kg | 24.93±1.16 | 10.71±0.44 | 5.99±0.22 | 4.41±0.14 |
| AKBA 40mg/kg | 24.87±1.60 | 10.74±0.82 | 6.04±0.54 | 4.54±0.36 |

The results shows that AKBA has no effect on Hemorheology at early stage of hypertensive rats. Mean ± SD (n=5), #P<0.05 vs. WKY group.

**Table S2.** Side effects assessment, renal function

| Groups | BUN (mM/L) | CRE (mM/L) |
| --- | --- | --- |
| WKY | 9.74±0.42 | 69.80±6.14 |
| SHR | 8.57±0.96 | 74.20±4.29 |
| AKBA 10mg/kg | 9.79±0.83 | 72.90±3.42 |
| AKBA 20mg/kg | 9.89±0.33 | 76.00±1.54 |
| AKBA 40mg/kg | 10.01±0.65 | 78.00±1.04 |

The results shows that at early stage of hypertension, BUN and CRE show no significant difference among groups, and AKBA treatment shows no notable side effects on renal function, mean ± SD (n=5).
